# Fullerene mixing effect on carrier formation in bulk-hetero organic solar cell

**DOI:** 10.1038/srep09483

**Published:** 2015-03-30

**Authors:** Yutaka Moritomo, Takeshi Yasuda, Kouhei Yonezawa, Takeaki Sakurai, Yasuo Takeichi, Hiroki Suga, Yoshio Takahashi, Nobuyuki Inami, Kazuhiko Mase, Kanta Ono

**Affiliations:** 1 Faculty of Pure and Applied Science, Univ. of Tsukuba, Tsukuba 305-8571, Japan; 2 Center for Integrated Research in Fundamental Science and Engineering (CiRfSE), Univ. of Tsukuba, Tsukuba 305-8571, Japan; 3 Photovoltaic Materials Unit, National Institute for Materials Science (NIMS), Tsukuba, Ibaraki 305-0047, Japan; 4 PRESTO, Japan Science and Technology Agency, Saitama 332-0012, Japan; 5 Institute of Materials Structure Science, High-Energy Accelerator Research Organization (KEK), Tsukuba, Ibaraki 305-0801, Japan; 6 Department of Earth and Planetary Systems Science, Hiroshima University, Higashi-hiroshima, Hiroshima 739-8526, Japan; 7 Department of Earth and Planetary Science, Univ. of Tokyo, Bunkyo-ku, Tokyo 113-0033, Japan

## Abstract

Organic solar cells (OSCs) with a bulk-heterojunction (BHJ) are promising energy conversion devices, because they are flexible and environmental-friendly, and can be fabricated by low-cost roll-to-roll process. Here, we systematically investigated the interrelations between photovoltaic properties and the domain morphology of the active layer in OSCs based on films of poly-(9,9-dioctylfluorene-co-bithiophene) (F8T2)/[6,6]-phenyl C_71_-butyric acid methyl ester (PC_71_BM) blend annealed at various temperatures (*T*_an_). The scanning transmission X-ray microscopy (STXM) revealed that fullerene mixing (Φ_Fullerene_) in the polymer matrix decreases with increase in *T*_an_ while the domain size (*L*) is nearly independent of *T*_an_. The TEM-S mapping image suggests that the polymer matrix consist of polymer clusters of several nm and fullerene. We found that the charge formation efficiency (Φ_CF_), internal quantum efficiency (Φ_IQ_), and power conversion efficiency (PCE) are dominantly determined by Φ_Fullerene_. We interpreted these observations in terms of the polymer clusters within the polymer matrix.

In OSCs, the light-to-electric energy conversion is realized by the combination of the carrier formation and transfer processes within the active layer (Fig. S1). In the former process, the photo irradiation creates a donor exciton in the donor region and the donor exciton migrates to the donor (D)/acceptor (A) interface. Finally, the exciton separates into the electron and hole at the D/A interface. In most cases, the electron and hole are weakly bound to each other around the interface. In the latter process, the carriers transfer to the collector electrode and are collected as photocurrent. This is in a sharp contrast with an inorganic solar cell (ISC), in which the photo-irradiation directly creates carriers within the active layer.

The BHJ active layer of OSC consists of phase-separated nano-size (several tens of nm) domains of the donor polymer and acceptor fullerene^1^,^2^,^3^,^4^,^5^. The nano-size structure is essential for the efficient carrier formation process, because the length of exciton migration is ~3 nm. The STXM around the carbon K-edge is a powerful tool to clarify the molecular mixing as well as the domain structure in the BHJ active layer^6^,^7^, because it can distinguish the fullerene carbon from the polymer carbon. For example, Collins *et al.*^6^ revealed the fullerene mixing in the polymer matrix of PTB7/PC_71_BM blend film. Due to the low spatial resolution (∼several tens of nm) of STXM, however, the domain size had to be enlarged by chemical admixture^6^ or thermal annealing at higher temperature^7^. Recently, several experiments revealed sub-structures within the large domains. By means of atomic force microscopy (AFM) coupled with plasma-ashing technique, Hedley *et al.*^8^ investigated sub-structure inside the domain (100–200 nm) of PTB7/PC_71_BM blend film prepared without additive and found that the domain consists of a large number of small fullerene spheres (20–60 nm). By means of energy-filtered electron transmission microscopy (EFTEM), Kesave *et al.*^9^ reported fiber-like structure, ~10 nm wide and ~100 nm long, in PGeBTBT/PC_71_BM blend film.

On the other hands, the femtosecond time-resolved spectroscopy is a powerful tool to reveal the carrier formation process within the active layer^10^,^11^,^12^,^13^,^14^, because the spectroscopy monitors the relative numbers of the photo-created exciton and carrier in the time domain. Significantly, the spectroscopy decouples the carrier formation and transfer processes, because the former process completes within several tens of ps. Actually, the time-resolved spectroscopy revealed that the exciton-to-carrier conversion process in PTB7/PC_71_BM blend film completes within ~0.3 ps^10^.

In order to clarify the interrelation between molecular mixing and the photovoltaic properties of BHJ-type OSCs, we selected a liquid-crystalline semiconducting polymer, F8T2, as the donor polymer, because the domain structure of the blend film with fullerene derivatives remains large (several hundreds of nm) and independent of *T*_an_^15^,^16^. Yasuda *et al*.^16^ systematically investigated the photovoltaic properties of the OSCs based on films of F8T2/PC_71_BM (33 : 67 wt. %) blend annealed at various temperature (*T*_an_): the PCE systematically decreases from the optimal value ( = 2.28%) at *T*_an_ = 80°C to 0.81% at 240°C. Yonezawa *et al*.^17^ investigated the charge formation dynamics of F8T2/PC_71_BM blend film by means of the femtosecond time-resolved spectroscopy. Here, we systematically investigated *T*_an_-dependence of the photovoltaic properties, *i.e.*, short-circuit current (*J*_sc_), open-circuit voltage (*V*_oc_), fill factor (FF), power conversion efficiency (PCE), internal quantum efficiency (Φ_IQ_), charge formation efficiency (Φ_CF_), domain size (*L*), and fullerene mixing (Φ_Fullerene_,) in the polymer matrix in the OSCs based on films of F8T2/PC_71_BM blend. Φ_CF_ is defined by n_formed_/n_photon_, where n_formed_ and n_photon_ are the numbers of the carriers formed at the D/A interface (include weakly bound state) and the absorbed photons, respectively. Absolute value of n_formed_ was estimated by combination of the time-resolved and electrochemical spectroscopies. Φ_CF_ is the same as the exciton quenching efficiency, if all the quenched excitons are converted to carriers. We found that Φ_CF_, Φ_IQ_, and PCE are dominantly determined by Φ_Fullerene_, indicating an essential role of the fullerene mixing in the polymer matrix on the carrier formation and transfer processes.

## Results

### Photovoltaic properties

First of all, let us survey the device parameter, *i.e.*, *J*_sc_, *V*_oc_, FF, PCE, and Φ_IQ_ against *T*_an_ (Table I). We fabricated OSCs based on films of F8T2/PC_71_BM (33 : 67 wt%) blend annealed for 10 min at *T*_an_ (Fig. S2). We measured current (*J*) – voltage (*V*) curve (Fig. S3) and incident photon-to-current conversion efficiency (IPCE) spectra (Fig. S4) .The magnitudes of *J*_sc_ and FF decrease with increase in *T*_an_, while *V*_oc_ remains nearly independent of *T*_an_. As a result, PCE ( = *J*_sc_ × *V*_oc_ × FF/*I*_0_, where *I*_0_ is the power density of the incident light) deceases with increase in *T*_an_. The *T*_an_-dependence of *J*_sc_ and FF is ascribed to several compounded factors, *e.g*., the domain size, carrier recombination process at the D/A interface, and connectivity among the domains. We confirmed that the domain size (*L*) of the active layered is nearly independent of *T*_an_ (*vide infra*).

### Domain structure as investigated by STXM image

**Figure 1:**
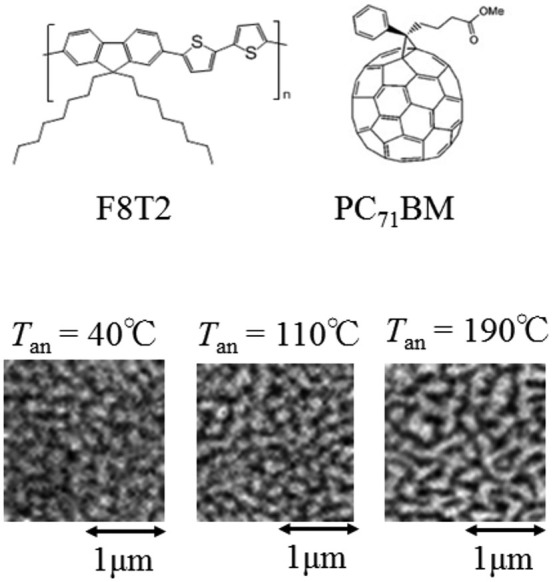
STXM image of F8T2/PC_71_BM blend films at 284.4 eV. The bright regions correspond to the PC_71_BM-rich domains, while the dark regions the F8T2-rich domains.

Figure 1 shows STXM images of the F8T2/PC_71_BM blend films annealed at various *T*_an_ probed at 284.4 eV. The photon energy (284.4 eV) was at the π*-resonance absorption of the fullerene framework^6^,^18^. Therefore, the bright regions correspond to the fullerene-rich domains, while the dark regions the polymer-rich domains. We performed two-dimensional Fourier transformation to evaluate the length scale (*L*) of the fullerene domain. We regarded the local maxima of the Fourier component as *L* (Fig. S5). We found that *L* (~270 nm) is nearly independent of *T*_an_.

### Fullerene mixing as investigated by STXM spectroscopy

To determine the fullerene mixing, we measured the carbon K-edge absorption spectra (φ_exp_) at every 40 nm within the 2 μm × 2 μm image, *i.e*., 50 × 50 spectra^19^. We should be careful for evaluation of the molecular mixing since the STXM spectra is average along the depth direction. We investigated cross-sectional Plasmon loss image (Fig. S6) of the blend film annealed at 80°C. We confirmed that the polymer matrix passes completely through to the other side. That is, the fullerene mixing of the polymer matrix is accurately evaluated by the STXM spectroscopy. Unfortunately, we cannot accurately evaluated the molecular mixing of the fullerene domain, because it overlaps with the polymer domain in the depth direction.

**Figure 2:**
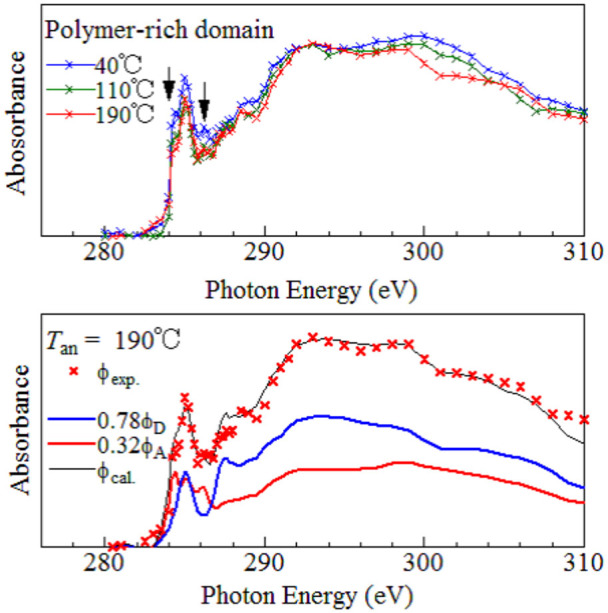
Carbon K-edge absorption spectra of the polymer matrix. Upper panel shows the averaged absorption spectra against *T*_an_. Lower panel shows an example of the spectral decomposition, which was performed by least-squares fitting of the observed spectra (φ_exp_) with the linear combination of the F8T2 (φ_D_) and PC_71_BM (φ_A_) spectra, φ_cal_ = C_D_φ_D_ + C_A_φ_A_.

Upper panel of Fig. 2 shows the averaged carbon K-edge absorption spectra of the polymer matrix against *T*_an_. We observed extra bands at both sides of the main peak at 285 eV, as indicated by downward arrows. The bands are ascribed to the 1^st^ and 3^rd^ peaks of PC_71_BM (see the lower panel of Fig. 2). Their intensities gradually increases with decreases in *T*_an_, indicating that the fullerene mixing increases with decreases in *T*_an_. The magnitudes of Φ_Fullerene_ were evaluated by least-squares fitting of the φ_exp_ spectra with the linear combination of the F8T2 (φ_D_) and PC_71_BM (φ_A_) spectra, φ_cal_ = C_D_φ_D_ + C_A_φ_A_. In the lower panel of Fig. 2, we show an example of the least-squares fitting. The linear combination (black thin curve) well reproduces the overall features of the φ_exp_ spectra. This indicates that the charge-transfer-type absorption at the D/A interface has negligible effects on the φ_exp_ spectra. In the spectral analysis, we select ten φ_exp_ spectra at every *T*_an_ at the central position of the polymer matrix to avoid the artificial mixing of the materials. The averages and standard deviations of C_D_ and C_A_ were evaluated. The Φ_Fullerene_ values were calculated by C_A_/(C_D_ + C_A_), and are listed in Table II.

### Carrier formation efficiency

**Figure 3:**
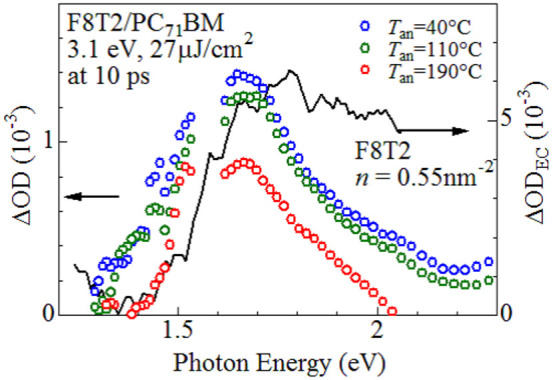
Differential absorption (ΔOD) spectra at 10 ps of F8T2/PC_71_BM blend film and differential absorption (ΔOD_EC_) spectrum of electrochemically oxidized F8T2 neat film. In ΔOD, the excitation photon energy and pulse energy are 3.1 eV and 27 μJ/cm^2^, respectively. In ΔOD_EC_, the hole-doing level (*n*) is 0.55 nm^−2^.

We evaluate the absolute value of Φ_CF_ by combination of the femtosecond time-resolved and electrochemical spectroscopies^20^. Solid curve in Fig. 3 is the differential absorption (ΔOD_EC_) spectrum of electrochemically oxidized F8T2 neat film. The observed absorption at 1.8 eV is ascribed to the donor carrier. We investigated the spectral intensity (*I*_1.8eV_) at 1.8 eV against the hole-doping level (*n*) and determined the coefficient (α_carrier_ = 4.1 × 10^−3^ nm^2^) between *I*_1.8eV_ and *n* (see Fig. S7). Circles in Fig. 3 is the differential absorption (ΔOD) spectra at 10 ps of the F8T2/PC_71_BM blend film. A sharp photoinduced absorption (PIA) is observed at 1.7 eV, whose profile is similar to that of the ΔOD_EC_ spectrum. We confirmed that the spectral profile is unchanged after 10 ps. In addition, the decay time of the PIA is very long ( = 300 ps). These observations indicate that the PIA is due to the donor carriers. We evaluated the coefficient (α_photon_) between the spectral intensity (*I*_1.7eV_) at 1.7 eV and the number (*n*_photon_) of the absorbed photons, with considering the reflection and transmission losses. The Φ_CF_ values were calculated by α_photon_/α_carrier_, and are listed in Table II.

### Correlation between parameters

**Figure 4:**
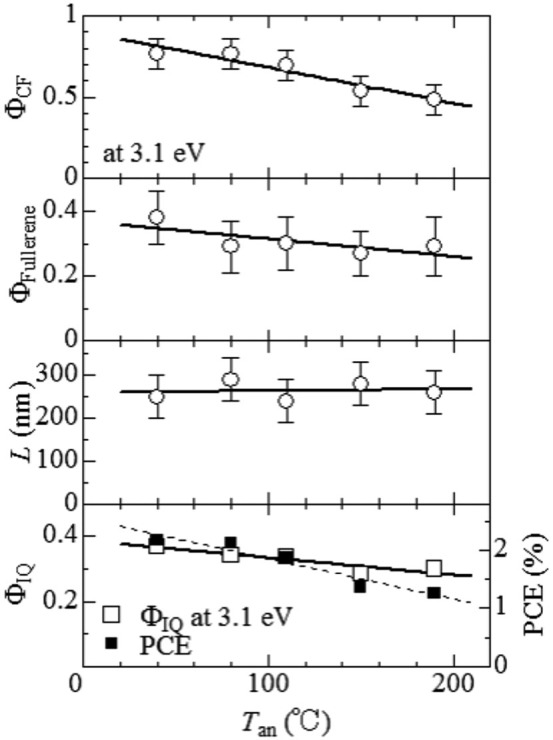
Interrelation among Φ_CF_, Φ_Fullerene_, *L*, Φ_IQ_, and PCE in OSCs against *T*_an_. Solid straight lines are the results of the lease-squares fittings. Error bars of Φ_IQ_, and PCE are within the symbol size.

We summarize in Fig. 4 the interrelation among Φ_CF_, Φ_Fullerene_, *L*, Φ_IQ_, and PCE in OSCs against *T*_an_. We note that Φ_CF_ has no relation with any losses after the carrier formation, *e.g*., the carrier recombination at the D/A interface or carrier trapping. In this sense, Φ_CF_ is easier to interpret than the other efficiencies such as Φ_IQ_, and PCE. Φ_CF_ systematically decreases from 0.78 to 0.48 with increase in *T*_an_. In the 2^nd^ and 3^rd^ panels, we plotted Φ_Fullerene_ and *L*. The *T*_an_ value has no effect on the domain size (*L* ~ 270 nm), but seems to suppress Φ_Fullerene_ in the polymer matrix. The *T*_an_-dependence of Φ_Fullerene_, however, is unclear due to rather large error bars. The error bars come from the bad signal/noise ratio in the featureless spectra. We carefully investigated *T*_an_-dependence of the spectral profile around the fullerene peaks (284–287 eV) in the averaged carbon K-edge absorption spectra. We found that the relative intensities (*I*_284.4eV_) of the fullerene peak at 284.4 eV systematically decreases with increase in *T*_an_ (Fig. S8). This observation indicates that the fullerene mixing in the polymer matrix decreases with increase in *T*_an_. Thus observed *T*_an_-dependence of the fullerene mixing is reasonable, because the thermal annealing at higher *T*_an_ accelerates the phase-separation into more pure domains. Our analysis revealed that the fullerene mixing in the polymer matrix is advantageous for the efficient carrier formation. The decrease in Φ_CF_ with *T*_an_ is responsible for the suppressed Φ_IQ_ and PCE (bottom panel of Fig. 4).

## Discussion

To investigate the morphology within the polymer matrix, we investigated cross-sectional TEM-S mapping image of the blend film annealed at 80°C (Fig. S9). The mapping clarifies the distribution of the F8T2 polymer in ∼ nm resolution. The mapping suggests that the F8T2 polymer matrix consists of the polymer clusters of several nm and the fullerene. Such a sub-structure well explains why the F8T2/PC_71_BM OSC shows high Φ_IQ_ ( = 0.35 at 40°C) even though its domain size (*L* ~ 270 nm) is too large for exciton to reach the domain boundaries. According to this scenario, our observation, *i. e.*, Φ_CF_, Φ_IQ_, and PCE decreases as the fullerene mixing in the polymer matrix decreases, is interpreted as follows. With increase in *T*_an_, the number (size) of the polymer clusters decreases (increases) within the polymer matrix, because the thermal annealing at higher *T*_an_ accelerates the phase-separation into more pure domains in every scale. As a result, the average fullerene concentration (Φ_Fullerene_) within the polymer matrix decreases with *T*_an_. The decrease in number and the increase in size of the polymer clusters lead to less donor exciton reaching to the D/A interface, to cause the suppressed Φ_CF_, Φ_IQ_, and PCE. The fullerene mixing is a two-edged blade, because the sub-structure interface also function as recombination point for the photo-generated carriers^21^. Here, we define the carrier transfer efficiency (Φ_CT_) as n_collected_/n_formed_, where n_collected_ is the number of the carriers collected as photocurrent (Fig. S1). Then, Φ_CT_ is evaluated by Φ_IQ_/Φ_CT_ (see Table II). We found that Φ_CT_ decreases with decrease in *T*_an_. The suppressed Φ_CT_ is ascribed to the enhanced carrier recombination process at the sub-structure interface.

## Summary

In summary, we systematically investigated the interrelations between photovoltaic properties, Φ_CF_, and Φ_Fullerene_ in OSCs based on films of F8T2/PC_71_BM blend annealed at various *T*_an_. We found that Φ_CF_, Φ_IQ_, and PCE are dominantly determined by *Φ*_Fullerene_, not by the size scale (*L*) of the domain. The TEM-S mapping image suggests that F8T2 polymer matrix consist of the polymer clusters of several nm and the fullerene. We interpreted the observation in terms of the polymer clusters within the polymer matrix: the decrease in number and the increase in size of polymer clusters lead to less donor exciton reaching to the D/A interface, to causes of the suppressed Φ_CF_, Φ_IQ_, and PCE. Even though the stability of the large-scale morphology against *T*_an_ is specific to the F8T2/PC_71_BM combination, the annealing effects on the sub-structure are considered to be general to the polymer/fullerene blend film. Thus, complementary study of STXM, which probes quantitative molecular mixing in several tens of nm scale, and TEM-S mapping, which probes molecular distribution in ∼ nm resolution, is effective to comprehend the photovoltaic properties of OSCs.

## Method

### Synthesis and characterization of the blend film

F8T2 was purchased from American Dye Source. The weight average molecular weight *M*_w_, number average molecular weight *M*_n_, and polydispersity *M*_w_/*M*_n_ were estimated to be 45,000, 13,000, and 3.4, respectively. PC_71_BM (purity 99%) was purchased from Solenne.

For the STXM measurements, F8T2/PC_71_BM blend films were transferred to a Si_3_N_4_ membrane. A bilayer film [poly(sodium 4-styrenesulfonate) (PSS)/blend film] was prepared by successive spin-coating of an aqueous solution of PSS and an *o*-dichlorobenzene (*o*-DCB) solution of F8T2/PC_71_BM (33 : 67 wt %). The thicknesses of the as-grown films were 71 nm. The films were annealed for 10 min at *T*_an_ = 40, 80, 110, 150, 190, and 240°C in a N_2_ glove box. Then, the bilayer film was cut into 1 × 1 mm^2^ pieces, and the substrate was immersed for several minutes in distilled water to etch away the PSS film. Thus, we obtained small F8T2/PC_71_BM films floating on the distilled water. A piece of the floating film was scooped up with the Si_3_N_4_ membrane (50 nm in thickness and 500 × 500 μm^2^ in area).

For the time-resolved spectroscopy, F8T2/PC_71_BM blend films were prepared by spin-coating of an *o*-DCB solution of F8T2/PC_71_BM (33 : 67 wt %) on quartz substrates. The thicknesses of the as-grown films were 60–70 nm. The films were annealed for 10 min at *T*_an_ = 40, 80, 110, 150, 190, and 240°C in a N_2_ glove box. The atomic force microscope (AFM) image of the blend film annealed below 190°C revealed a periodic nanostructure of 300 nm in diameter (Fig. S2). The blend film annealed at 240°C is known to show macro-scale phase-separation into pure-F8T2 and pure-PC_71_BM domains.

### Fabrication and characterization of the OSC

The OSCs were fabricated with a structure of indium tin oxide (ITO)/poly(3,4-ethylenedioxythiophene) (PEDOT):PSS (40 nm)/blend film/LiF (1 nm)/Al (80 nm). The patterned ITO (conductivity: 10 Ω/square) glass was pre-cleaned in an ultrasonic bath of acetone and ethanol and then treated in an ultraviolet-ozone chamber. A thin layer (40 nm) of PEDOT:PSS was spin-coated onto the ITO and dried in air at 110°C for 10 min on a hot plate. The substrate was then transferred to an N_2_ glove box and dried again at 110°C for 10 min on a hot plate. An *o*-DCB solution of F8T2:PC_71_BM (33 : 67 wt %) was subsequently spin-coated onto the PEDOT:PSS surface to form the active layer. The resultant substrates were then annealed at *T*_an_ = 40, 80, 110, 150, 190, and 240°C for 10 min in a N_2_ glove box. Finally, LiF (1 nm) and Al (80 nm) were deposited onto the active layer by conventional thermal evaporation at a chamber pressure lower than 5 × 10^−4^ Pa. The active area of the OSCs is 2 × 2 mm^2^. The *J* - *V* curves (see Fig. S3) were measured using a voltage - current source/monitor under AM 1.5 solar-simulated light irradiation of 100 mW/cm^2^ (Bunkou-keiki, OTENTO-SUN III). The IPCE spectra (see Fig. S4) was measured using a SM-250 system (Bunkou-keiki). The internal quantum efficiencies (Φ_IQ_) at 400 nm were estimated with considering the reflection loss.

### STXM spectroscopy and analysis

The STXM measurement was performed using the compact STXM installed at the BL-13A beamline of the Photon Factory (PF), KEK. The details of the compact STXM are described in the literature^19^. The spatial resolution was 30–40 nm. The carbon K-edge absorption spectra (φ_exp_) were measured at every 40 nm within the 2 μm × 2 μm image, *i.e*., 50 × 50 spectra. The molecular mixing was evaluated by least-squares fitting of the observed spectra (φ_exp_) with the linear combination of the F8T2 (φ_D_) and PC_71_BM (φ_A_) spectra, φ_cal_ = C_D_φ_D_ + C_A_φ_A_. We regard the absorption spectra of the F8T2 and PC_71_BM domains in the F8T2/PC_71_BM blend film annealed at 240°C as φ_D_ and φ_A_, respectively. The coefficients, C_A_ and C_D_, are determined so that the evaluation function, 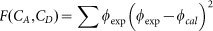, becomes the minimum. The background constant component was subtracted so that φ_cal_ becomes zero at 280 eV. In the spectral analysis, we select the ten φ_exp_ spectra at the central position of the polymer matrix to avoid the artificial mixing of the materials. The averages and standard deviations of C_D_ and C_A_ were evaluated. The volume fractions of fullerene (Φ_Fullerene_) were calculated by C_A_/(C_D_ + C_A_).

### Femtosecond time-resolved spectroscopy

The time-resolved spectroscopy was performed in a pump-probe configuration. In order to reduce the irradiation damage, the blend films were placed in N_2_ atmosphere. The pump pulse at 400 nm was generated as the second harmonics of a regenerative amplified Ti: sapphire laser in a β-BaB_2_O_4_ (BBO) crystal. The pulse width, repetition rate, and pulse energy were 100 fs, 1000 Hz, and 27 μJ/cm^2^ respectively. The frequency of the pump pulse was decreased by half (500 Hz) to provide “pump-on” and “pump-off” conditions. A white probe pulse (500–900 nm), generated by self-phase modulation in a sapphire plate was focused on the sample with the pump pulse. The spot sizes of the pump and probe pulses were 4.0 and 2.0 mm in diameter, respectively. The differential absorption (ΔOD) spectrum is expressed as −log(*I*_on_/*I*_off_), where *I*_on_ and *I*_off_ are the transmission spectra under the pump-on and pump-off conditions, respectively.

### Electrochemical spectroscopy

The electrochemical spectroscopy was carried out in an optical two-pole cell with a pair of quartz windows. The electrochemical hole-doping was performed against Li metal in propylene carbonate (PC) solution containing 1 mol/L LiClO_4_. The F8T2 neat film was spin-coated on an ITO glass substrate from *o*-DCB solution, and was dried in a N_2_ glove box. The thicknesses was 67 nm. The active area of the film was 2.25 cm^2^, and the reduction current was 100 nA. The voltage in the hole-doping process were 3.8 V vs. Li. The differential absorption (ΔOD_EC_) spectrum of electrochemically oxidized film is expressed as −log(*I*_doped_/*I*_non_), where *I*_doped_ and *I*_non_ are the transmission spectra of the hole-doped and non-doped films, respectively.

The charge formation efficiency (Φ_CF_) was determined by combination of the time-resolved and electrochemical spectroscopies^20^. The former spectroscopy tells us the coefficient (α_photon_) between ΔOD and *n*_photon_, while the latter spectroscopy tells us the coefficient (α_carrier_) between ΔOD_EC_ and *n*. Then, the Φ_CF_ value is calculated by α_photon_/α_carrier_.

## Supplementary Material

Supplementary Information Supplementary information

## Figures and Tables

**Table 1:** Device parameters, *i.e.*, *J*_sc_, *V*_oc_, FF, PCE and Φ_IQ_ of OSCs based on films of F8T2/PC_71_BM (33 : 67 wt%) blend annealed for 10 min at *T*_an_. The error bars of *J*_sc_, *V*_oc_, FF, PCE, and Φ_IQ_ were estimated from standard deviations of more than five OSC devices

*T*_an_ (°C)	*J*_sc_ (mA/cm^2^)	*V*_oc_ (V)	FF	PCE (%)	Φ_IQ_ @ 400 nm
40	4.37 ± 0.08	0.96 ± 0.01	0.516 ± 0.003	2.17 ± 0.04	0.368 ± 0.011
80	4.17 ± 0.08	0.97 ± 0.01	0.521 ± 0.003	2.12 ± 0.04	0.342 ± 0.009
110	3.96 ± 0.17	0.987 ± 0.006	0.486 ± 0.002	1.92 ± 0.09	0.336 ± 0.009
150	3.68 ± 0.19	0.94 ± 0.02	0.40 ± 0.01	1.37 ± 0.01	0.285 ± 0.007
190	3.51 ± 0.10	0.92 ± 0.01	0.387 ± 0.003	1.25 ± 0.05	0.300 ± 0.010
240	2.94 ± 0.17	0.91 ± 0.01	0.35 ± 0.01	0.95 ± 0.06	0.310 ± 0.015

**Table 2:** Internal quantum efficiency (Φ_IQ_), carrier formation efficiency (Φ_CF_), carrier transfer efficiency (Φ_CT_ = Φ_IQ_/Φ_CF_), and fullerene mixing (Φ_Fullerene_) within the polymer matrix of OSCs based on films of F8T2/PC_71_BM (33 : 67 wt%) blend annealed for 10 min at *T*_an_. The error bars of Φ_CF_ were roughly evaluated from the signal/noise ratio of the femtosecond time-resolved spectra. Φ_Fullerene_ was evaluated by least-squares fitting of the observed spectra (φ_exp_) with the linear combination of the F8T2 (φ_D_) and PC_71_BM (φ_A_) spectra, φ_cal_ = C_D_φ_D_ + C_A_φ_A_. The averages and standard deviations of C_D_ and C_A_ were evaluated from ten φ_exp_ spectra at every *T*_an_. The Φ_Fullerene_ values were calculated by C_A_/(C_D_ + C_A_)

*T*_an_ (°C)	Φ_IQ_ @ 400 nm	Φ_CF_ @ 400 nm	Φ_CT_ @ 400 nm	C_D_	C_A_	Φ_Fullerene_
40	0.368 ± 0.011	0.76 ± 0.04	0.48 ± 0.04	0.75 ± 0.07	0.47 ± 0.05	0.38 ± 0.08
80	0.342 ± 0.009	0.76 ± 0.04	0.45 ± 0.04	0.85 ± 0.05	0.36 ± 0.06	0.29 ± 0.08
110	0.336 ± 0.009	0.69 ± 0.04	0.49 ± 0.04	0.84 ± 0.04	0.36 ± 0.04	0.30 ± 0.05
150	0.285 ± 0.007	0.53 ± 0.04	0.53 ± 0.05	0.90 ± 0.07	0.33 ± 0.05	0.27 ± 0.07
190	0.300 ± 0.010	0.48 ± 0.04	0.63 ± 0.07	0.76 ± 0.03	0.31 ± 0.06	0.29 ± 0.09
240	0.310 ± 0.015	0.59 ± 0.04	0.53 ± 0.06	--------	--------	--------
