# Supplementary material for: Fullerene mixing effect on carrier formation in bulk-hetero organic solar cell
Source: Sci Rep. 2015 Mar 30;5:9483. doi: 10.1038/srep09483 (PMC5380156; doi:10.1038/srep09483)
Supplement: Supplementary Information [file srep09483-s1.pdf]

Supporting information

Fullerene mixing effect on carrier formation in bulk-hetero organic solar cell

Yutaka Moritomo[1,2]*, Takeshi Yasuda[3], Kouhei Yonezawa[1], Takeaki Sakurai[1,4], Yasuo Takeichi[5], Hiroki Suga[6], Yoshio Takahashi[5,7], Nobuyuki Inami[5], Kazuhiko Mase[5], and Kanta Ono[5]

[1]Fucalty of Pure and Applied Science, Univ. of Tsukuba, Tsukuba 305-8571, Japan

[2]Center for Integrated Research in Fundamental Science and Engineering (CiRfSE), Univ. of Tsukuba, Tsukuba 305-8571, Japan

[3]Photovoltaic Materials Unit, National Institute for Materials Science (NIMS), Tsukuba, Ibaraki 305-0047, Japan

[4]PRESTO, Japan Science and Technology Agency, Saitama 332-0012, Japan

[5]Institute of Materials Structure Science, High-Energy Accelerator Research Organization (KEK), Tsukuba, Ibaraki 305-0801, Japan

[6]Department of Earth and Planetary Systems Science, Hiroshima University, Higashi-hiroshima, Hiroshima 739-8526, Japan

[7]Department of Earth and Planetary Science, Univ. of Tokyo, Bunkyo-ku, Tokyo 113-0033, Japan

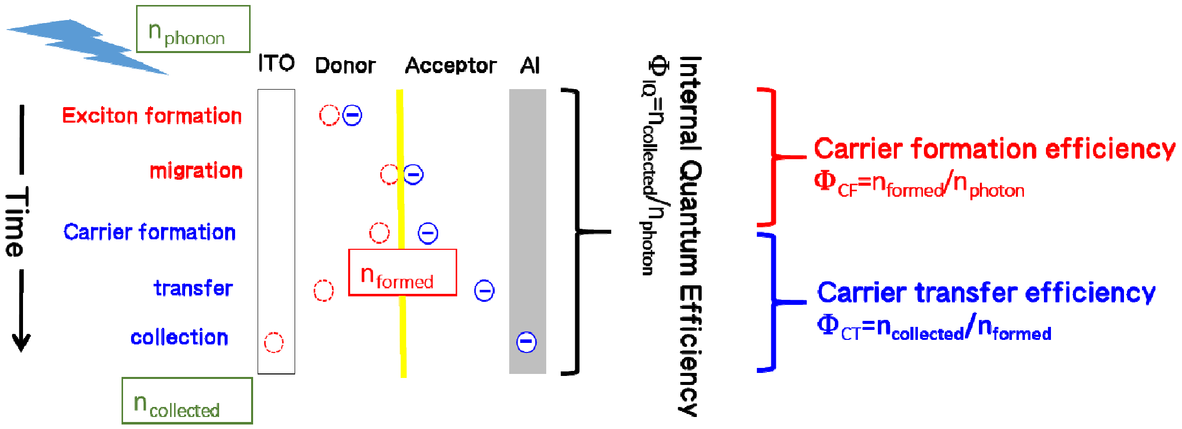

Fig. S1: Schematic illustration of photovoltaic process of an OSC. The light-to-electric energy conversion is realized by the carrier formation and transfer processes. In the former process, the photo irradiation creates a donor exciton in the donor region, and the donor exciton migrates to the D/A interface, and the exciton separates into electron and hole at the interface. In most cases, the electron and hole are weakly bound to each other around the interface. In the latter process, the carriers transfer to the collector electrode and are collected as photocurrent. The internal quantum efficiency (Φ_{IQ}), carrier formation efficiency (Φ_{CF}), and carrier transfer efficiency (Φ_{CT}) are defined by $\Phi_{IQ} = n_{collected} / n_{photon}$, $\Phi_{CF} = n_{formed} / n_{photon}$, and $\Phi_{CT} = n_{collected} / n_{formed}$, respectively. n_{photon}, n_{formed}, and $n_{collected}$ are the numbers of the absorbed photons, the carriers formed at the interface (vertical yellow line), and the carriers collected as current, respectively.

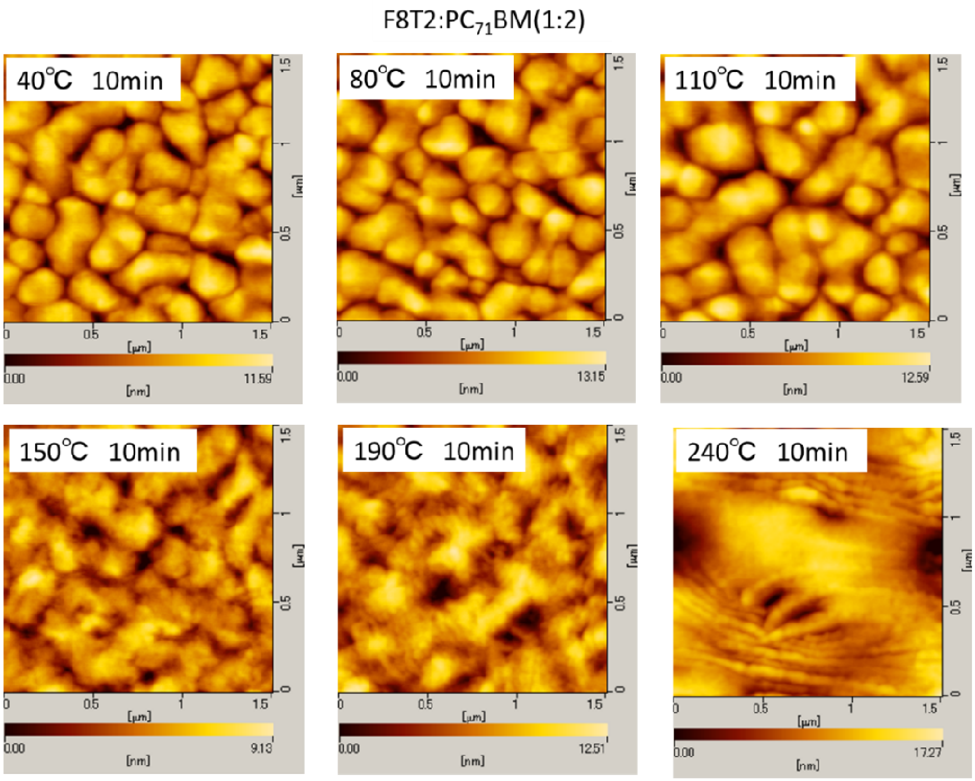

Fig S2: AFM image of F8T2/$PC_{71}BM$ (33 : 67 wt %) blend film after annealing for 10 min at various T_{an}. The blend films were spin-coated on PSS.

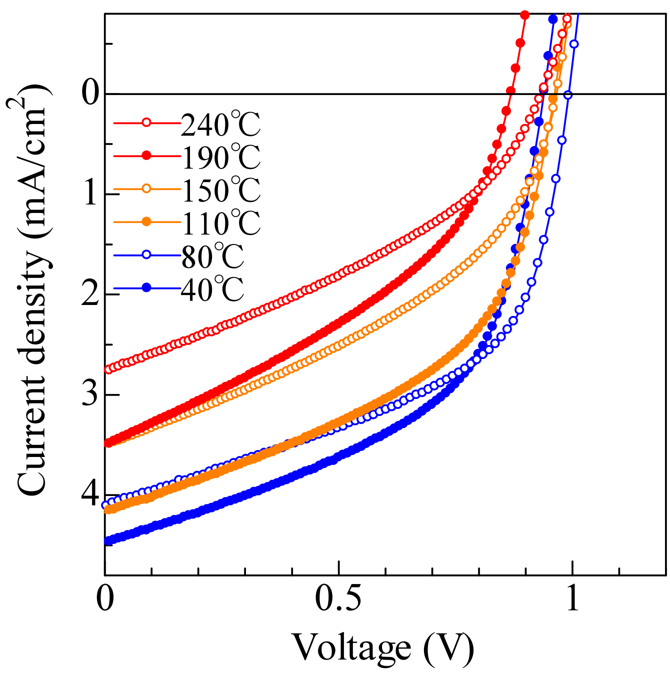

Fig. S3: Current density – voltage curves of OSCs based on films of F8T2/$PC_{71}BM$ (33 : 67 wt%) blend annealed for 10 min at various T_{an}. The OSC configuration is ITO/PEDOT:PSS (40 nm)/blend film/LiF (1 nm)/Al (80 nm).

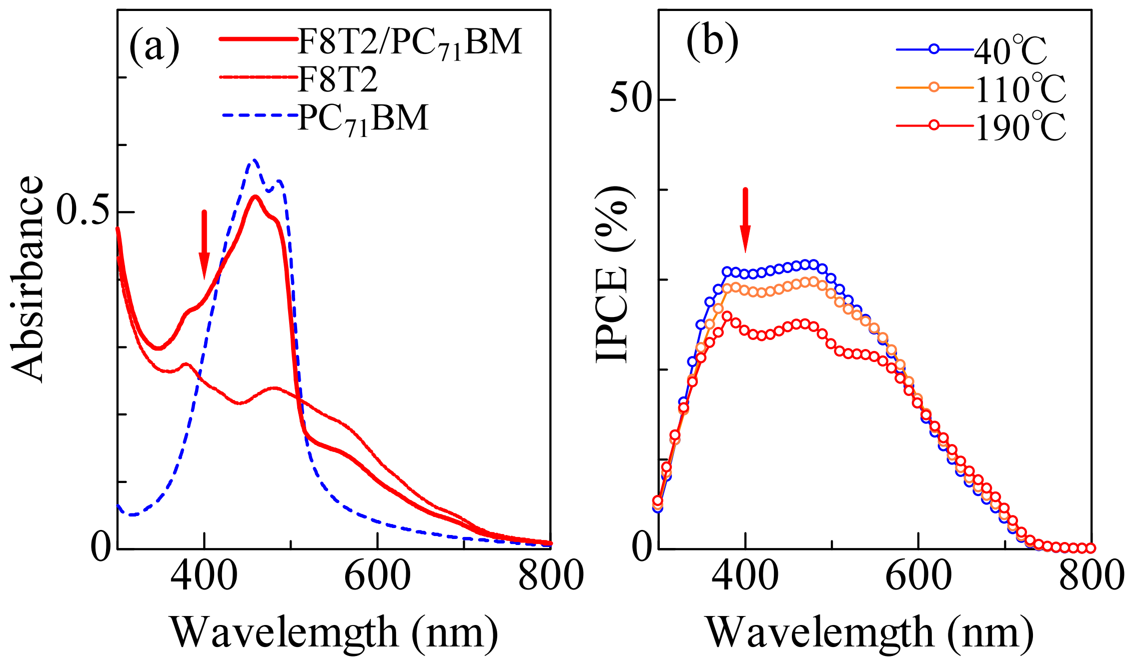

Fig. S4: (a) Absorption spectra of F8T2/$PC_{71}BM$ (65 nm), blend F8T2 neat (36 nm), and $PC_{71}BM$ (29 nm) neat films. (b) Incident photon-to-current conversion efficiency (IPCE) of OSCs based on films of F8T2/$PC_{71}BM$ (33 : 67 wt%) blend annealed for 10 min at T_{an}. The OSC configuration is ITO/PEDOT:PSS (40 nm)/blend film/LiF (1 nm)/Al (80 nm). Downward arrows indicate the excitation wavelength for the time-resolved spectroscopy.

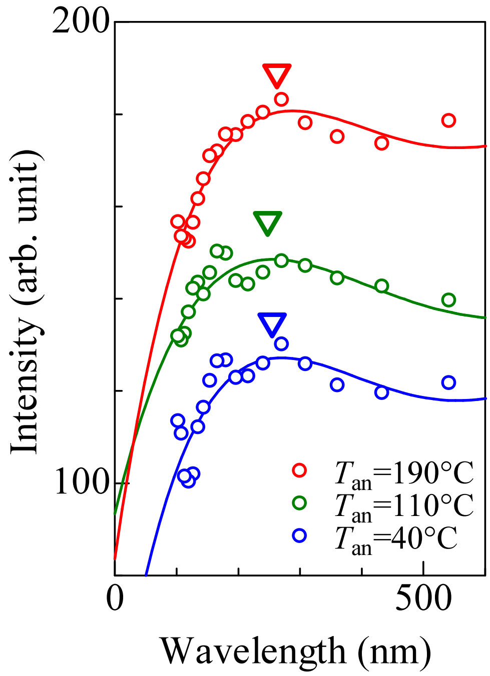

Fig. S5: Intensity of the two-dimensional Fourier transformation of the fullerene image against the wavelength. We regarded the local maxima (indicated by triangles) as the length scale (*L*) of the domain structure.

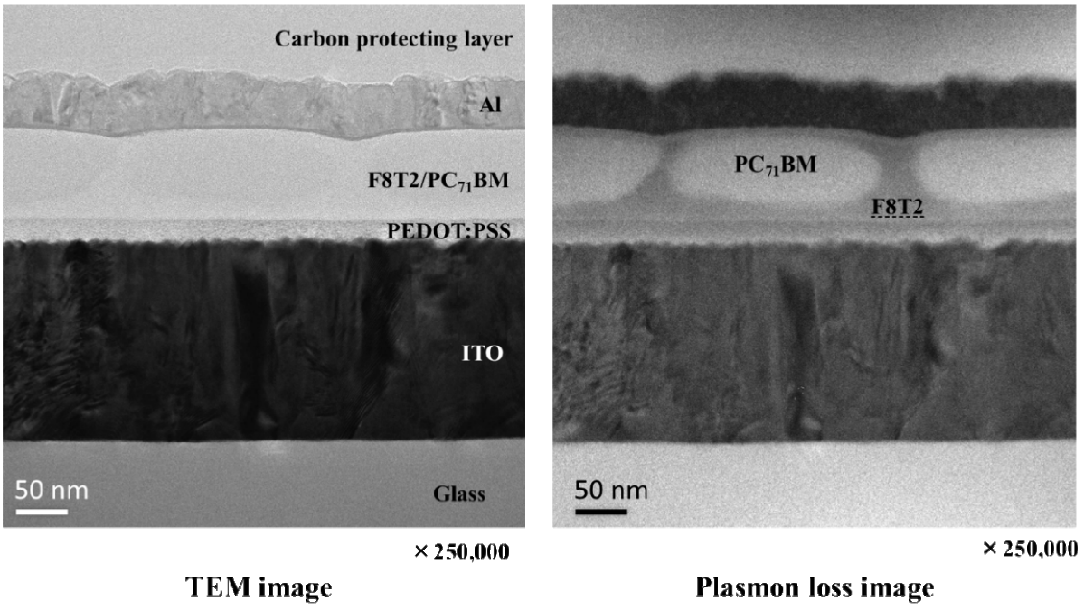

Fig. S6: Transmission electron microscopy (TEM) image (left panel) and Plasmon loss image (right panel) of OSC based on film of F8T2/$PC_{71}BM$ (33 : 67 wt%) blend annealed for 10 min at 80°C. The fullerene domains (bright region in the right panel) disperse within the polymer matrix (dark region). The polymer matrix passes completely through to the other side.

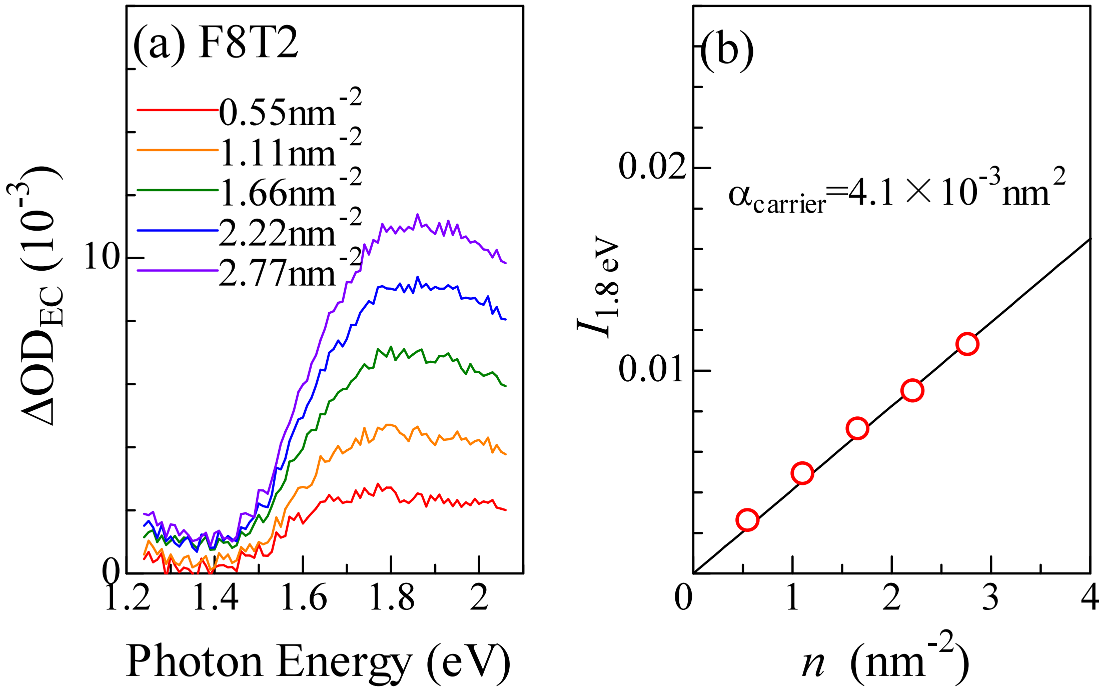

Fig. S7: (a) Differential absorption spectra (ΔOD_{EC}) of electrochemically oxidized F8T2 neat film. (b) Spectral intensity ($I_{1.8\ eV}$) at 1.8 eV against hole-doping level (n). The straight line is a result of the least-squares fitting.

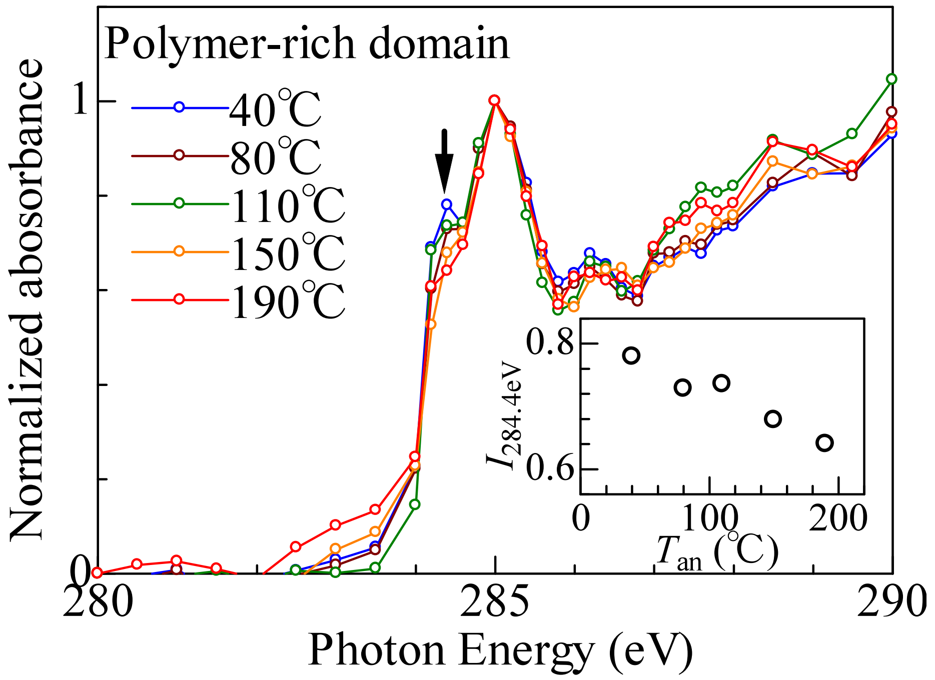

Fig. S8: Averaged carbon K-edge absorption spectra of the polymer matrix against T_{an}. The spectra are normalized at 285.0 eV. A downward arrow indicates the fullerene peak. Inset shows the intensity ($I_{284.4eV}$) of the fullerene band at 284.4 eV against T_{an}. The $I_{284.4eV}$ value decreases with increase in T_{an}.

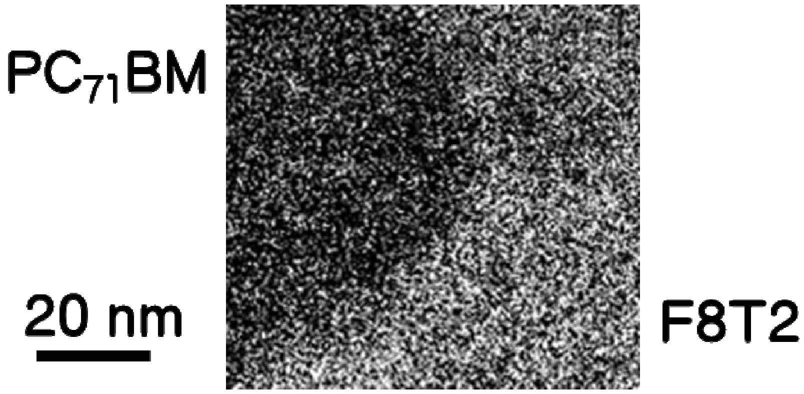

TEM-S mapping image

Fig. S9: TEM-S mapping image of OSC based on film of F8T2/$PC_{71}BM$ (33 : 67 wt%) blend annealed for 10 min at 80°C. The F8T2 polymer matrix consists of the polymer clusters of several nm and the fullerene.
